# MicroRNAs: short non-coding players in cancer chemoresistance

**DOI:** 10.1186/2052-8426-2-16

**Published:** 2014-06-01

**Authors:** Sara Donzelli, Federica Mori, Francesca Biagioni, Teresa Bellissimo, Claudio Pulito, Paola Muti, Sabrina Strano, Giovanni Blandino

**Affiliations:** Translational Oncogenomics Unit, Italian National Cancer Institute ‘Regina Elena’, Via Elio Chianesi 53, 00144 Rome, Italy; Molecular Chemoprevention Unit, Italian National Cancer Institute ‘Regina Elena’, Rome, Italy; Department of Oncology, Juravinski Cancer Center-McMaster University, Hamilton, Ontario Canada; College of Agriculture and Environmental Sciences, Unisa, Florida campus, Johannesburg, South Africa

**Keywords:** Chemoresistance, MicroRNA, Multiple drug resistance

## Abstract

Chemoresistance is one of the main problems in the therapy of cancer. There are a number of different molecular mechanisms through which a cancer cell acquires resistance to a specific treatment, such as alterations in drug uptake, drug metabolism and drug targets. There are several lines of evidence showing that miRNAs are involved in drug sensitivity of cancer cells in different tumor types and by different treatments. In this review, we provide an overview of the more recent and significant findings on the role of miRNAs in cancer cell drug resistance. In particular, we focus on specific miRNA mechanisms of action that in various steps lead from drug cell sensitivity to drug cell resistance. We also provide evidence on how miRNA profiling may unveil relevant predictive biomarkers for therapy outcomes.

## Review

### Introduction

Cancer is one of the leading causes of death worldwide where significant effort in searching for more effective therapies are relentlessly taking place. Chemotherapy, in synergy with radiotherapy and surgery, are the approaches commonly used for cancer treatment.

The main problem in cancer therapy is the frequent occurrence of resistance to different chemotherapeutic drugs that allows cancer cells to proliferate uncontrollably and to become more aggressive with a greater ability to metastasize to other organs.

Tumors can display either an intrinsic chemoresistance being originally insensitive to the treatment, or an acquired chemoresistance, being responsive at the beginning and then later becoming refractory to the cure [1]. In both cases the result is an inefficient intervention with consequent worsening of the disease.

Different mechanisms of cell chemoresistance have been described in the last few decades and can be roughly categorised in three main groups: 1) impairment in drug concentration due to either decreasing in drug influx or increasing in drug efflux; 2) alteration in drug-target connection; and 3) modification in cell-cycle and checkpoint mediators. An important feature in the field of chemoresistance is that a cancer cell may not only possibly be or become resistant to one drug, but also to different drugs. This latter phenomenon is known as multidrug resistance (MDR).

It has become vital to find the underlying molecular mechanisms involved in the drug resistance process to identify its determinants and regulators with the aim to discover specific new compounds able to interfere with that process.

Recent and numerous studies have pointed out the importance of microRNAs (miRNAs) in regulating many biological processes among which the regulation of tumor cell sensitivity to drugs.

MiRNAs are a family of small non-coding RNAs of 20–25 nucleotide length that are evolutionarily conserved across species and regulate gene expression at the post-transcriptional level [2, 3].

**Figure 1:**
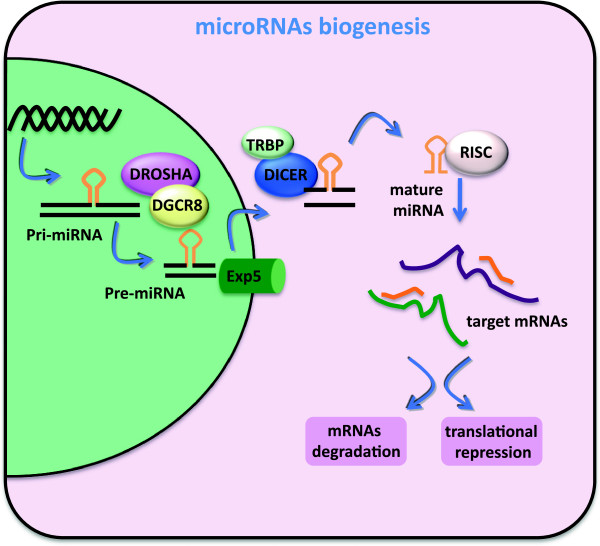
**The biogenesis of miRNAs.** RNA polymerase II transcribes miRNA gene, generating a long primary transcript (pri miRNA). Drosha-mediated cleavage of pri-miRNA leads to the formation of a hairpin molecule, the pre-microRNA that is exported to the cytoplasm by the complex exportin-5/RAN-GTP. In the cytoplasm, Dicer cleaves the pre-miRNA and produces a ~22-nt RNA duplex. The functional strand of the duplex is incorporated into a multiple-protein nuclease complex, the RNA-induced silencing complex (RISC), which regulates protein expression.

MiRNA biogenesis occurs in two different steps (Figure 1). In the nucleus, RNA Polymerase II transcribes miRNA genes in 170 bp primary transcripts (pri-miRNA) that contain both a 5’cap and a poly (A) tail and fold into a hairpin-shaped structure [4]. The Nuclear RNase III Drosha in cooperation with DGCR8 cofactor recognize and cleave these precursor molecules into a 70 nucleotide pre-miRNA [5]. Pre-miRNA is then exported in the cytoplasm through a process involving Exportin5 protein that is able to recognize a protruding tail of 2–3 nucleotides at 3’-end of the hairpin and promotes translocation into the cytoplasm [6, 7]. In the cytoplasm the RNAse Dicer binds and processes pre-miRNA in the transitory 22 bp miRNA-3p/miRNA-5p duplex. Within this process TRBP (TAR RNA-binding protein) and Ago2 assist Dicer as part of the RISC Loading Complex [8, 9]. One strand of this RNA duplex, the mature miRNA, enters into the miRNA–induced silencing complex (miRISC). Once in the miRISC, partial pairing between a miRNA and the 3’UTR of target mRNA results either in the repression of protein translation or, in case of complete pairing, in mRNA degradation [10].

MiRNAs regulate more than 50% of human transcriptome and thereby control the global activity of the cell. Indeed, miRNAs affect most cellular processes such as cell proliferation, differentiation, apoptosis, stress response, metabolism and angiogenesis. Therefore, it is not surprising that aberrant expression of miRNAs can be associated to human pathologies, including cancer [11].

Due to the small size and stability of miRNAs molecules they can be transferred from one cell to another one in different ways. Different studies have shown the transfer of miRNAs among cancer cells and between breast cancer cells and bone marrow stroma. This occurs through the gap junctions [12]. There is also evidence showing exosome-mediated directed transfer of specific miRNAs between cells. Montecalvo et al., reported that dendritic cells (DCs) secrete exosomes loaded with distinct sets of miRNAs that are related to the status of DC activation [13].

The role of miRNAs in the control of cancer cell chemoresistance has been confirmed by different studies highlighting how the deregulation of miRNAs expression can lead to a chemoresistant phenotype in different tumor types [14]. Therefore, miRNA activity could impinge on drug effects by modulating the expression of genes involved in the apoptotic response, in the DNA repair or in the control of cell cycle. MiRNA can also be under the control of different tumor suppressor genes or oncogenes, thus they might consequently be the intermediate effectors of a specific response to treatments. MiRNAs may also modulate the expression of genes involved in drug transport or metabolism. There is now increasing evidence that miRNAs can be predictor markers of chemotherapeutic response.

Below, we describe most of the different aspects of miRNA involvement in anticancer treatment response (see Table 1).

**Table 1:** **miRNAs involved in chemoresistance**

Pathway	microRNA	Target gene	Effect on chemoresistance	Cancer type	Reference
**APOPTOSIS**	miR-125b	p53 Bak	Resistance to doxorubicin, vincristin, etoposide, mafosfamide	Ewing sarcoma/pri itive neuroectodermal tumor	[24]
	miR-34a	SIRT1	Sensitivity to camptothecin	Prostate cancer	[27]
	miR-128-2	E2F5	Resistance to cisplatin, doxorubicin, 5-fluorouracile	Lung cancer	[39]
	miR-223	STMN-1	Sensitivity to cisplatin, 5-fluorouracil	Breast cancer, colon cancer	[40]
	miR-21, miR-221, miR-214	PTEN	Resistance to gemcitabine, cisplatin, gefitinib	Malignant human cholangiocytes, gastric cancer osteosarcoma, ovarian cancer, lung cancer	[42–48]
**DNA REPAIR**	miR-155, miR-21	MSH2, MSH6	Resistance to 5‒fluorouracil	Colorectal cancer	[16, 17]
	miR-138	H2AX	Sensitivity to cisplatin, 5-fluorouracil	Osteosarcoma	[18]
	miR-18a	ATM	Sensitivity to etoposide	Colorectal cancer	[19]
	miR-182	BRAC1	Sensitivity to PARP1 inhibitor	Breast cancer	[21]
**MDR**	miR-298, miR-27a, miR‒331‒5p	P-gp	Sensitivity to doxorubicin	Breast cancer, leukaemia	[66, 69]
	miR-19a/b,	PTEN	Resistance to cisplatin, doxorubicin, 5-fluorouracil	Gastric cancer	[67]
	miR-451	-	Resistance to vinblastin	Ovarian cancer	[68]
	miR-326	MRP1	Sensitivity to VP-16	Breast cancer	[70]
	miR-520 h, miR-519c, miR‒328	BCRP	Sensitivity to mitoxantrone	Hematopoietic stem cell, lung cancer, breast cancer, colon cancer	[71–73]
**DRUG METABOLISM**	miR-27b	CYP1B1	Induction	Breast cancer	[74]
	miR-148a	CYP34A	Induction	Liver cancer	[75]

### miRNAs as modulators of drug effects

#### Modulation in DNA-repair pathways

Chemotherapeutic drugs act in tumor cells affecting different cellular functions and causing different cellular responses. DNA damage response is the first reaction to genotoxic injury. Beyond the importance of this machinery for the integrity of the cells, alterations in the components of the DNA repair machinery is associated with chemoresistance. This is specifically evident in the case of platinum agents and alkylating compounds [15]. There are three fundamental pathways to repair damaged DNA: Nuclear excision repair (NER), Base excision repair (BER) and DNA mismatch repair (MMR) and all of them are, at different levels, involved in the chemoresistance.

Several recent studies have demonstrated how oncogenic miRNAs may interfere with DNA-repair pathways allowing cells to resist drugs that initially were effective against them. One example of this resistance is miR-155 which significantly down-regulates the mismatch repair recognition protein complex MSH2-MSH6 and MLH1-PMS2 [16]. Also miR-21 has been shown to contribute to chemoresistance by interfering with the DNA-repair system. Valeri and collaborators demonstrated that miR-21 targets MSH2 and MSH6 proteins determining an increased resistance to 5-fluorouracil treatment in colorectal cancer [17].

One of the initial signs of DNA damage is the phosphorylation of the histone H2A variant H2AX (γ-H2AX) by the ATM/ATR signalling pathway. Wang and colleagues identified miR-138 as a direct inhibitor of H2AX expression [18]. The authors initially performed a cell-based miRNA library screening, using IR-induced γH2AX foci formation as readout. From this experiment they obtained a list of miRNAs that regulated γH2AX foci formation. Then they focused their attention on miR-138, which had the most robust effect on γH2AX transcript. By further investigations they also demonstrated that overexpression of miR-138 sensitized tumor cells to DNA damaging agents such as cisplatin and camptothecin.

ATM activation represents an early and important event for DNA repair upon DNA double strand breaks. Wu and colleagues identified mir-18a which directly binds to ATM 3’UTR inducing increased sensitivity of colorectal cancer cells to etoposide [19].

Moreover, it was recently demonstrated that in triple negative breast cancers, miR-181 a/b are negative regulators of ATM expression and that these two miRNAs sensitize cancer cells to poly-ADP-ribose-polymerase 1 (PARP1) treatments [20].

Moskwa and collaborators showed that miR-182 expression impacts on BRCA1 protein expression in different breast cancer cell lines, determining its downregulation and a consequent increase in sensitivity to PARP1 inhibitor either in cultured cells or in xenograft models [21].

#### Alterations in apoptotic pathways

After DNA damage induced by anticancer drugs, injured cells can react in two ways: either by cell cycle arrest and damage repair or, if the problem is far too extensive to repair, cell death and apoptosis.

The final goal of cancer treatment is the death of the transformed cells. Defects in the apoptotic machinery have been described to play an important role in drug resistance [22].

Some miRNAs involved in chemoresistance act at specific steps in this pathway influencing the balance towards cell survival despite cell death.

The tumor suppressor protein p53 is a central player in the regulation of cell cycle arrest and cell death, and it is activated in response to different chemotherapeutic drugs [23].

Recently, Iida and colleagues demonstrated that miR-125b induces chemoresistance to doxorubicine, vincristine, etoposide and mafosfamide in Ewing sarcoma/primitive neuroectodermal tumor (EWS) by suppressing p53-dependent apoptosis [24]. In particular, they observed higher levels of miR-125b in doxorubicin-resistant EWS cells (VH-64/ADR) by comparing miR-125b expression in the parental VH-64 cells; when miR-125b was stably downregulated, an increase in caspase 3 cleavage, along with enhanced cell death upon doxorubicin treatment were evidenced. They also observed that p53 and Bak genes are among the putative miR-125b target mRNAs, thereby showing that miR-125b-mediateted chemoresistance is due to its ability to target p53 and Bak expression.

Likewise, miR-140 has been shown to interfere with p53-mediated apoptosis. Song and colleagues demonstrated that miR-140 promotes chemoresistance only in a wild type p53 cellular context. The chemoresistant effect of miR-140 is due to its ability in reducing cell proliferation and in inducing cell cycle arrest protecting cells from chemotherapy-induced damage. This occurred mainly by the reduction of HDAC4 protein expression only in p53 wild type cells.

p53 protein itself has been shown to modulate miRNAs expression and to induce cell cycle arrest and apoptosis: miR-34a is one of p53 effector genes [25]. Fujita and colleagues analyzed miR-34a expression in different prostate cancer cell lines with different p53 protein status (null, mutated or wild-type) observing a strong linear correlation between its expression and p53 wild-type expression [26]. They also demonstrated that ectopic expression of miR-34a in p53-null or p53-mutated prostate cancer cells restores chemosensitivity to camptothecin by suppression of SIRT1 and consequent induction of cell cycle arrest and apoptosis.

miR-122 was demonstrated to be a positive regulator of p53 protein stability able to contribute to chemosensitivity. One of the miR-122 target genes is cyclin G1, a well known negative regulator of p53 protein stability [27, 28]. Moreover, it has been shown that miR-122 overexpression in HCC cells restores sensitivity to doxorubicin treatment [27].

TP53 gene mutations are the most frequent event in human cancers. They are mainly missense mutations in the exons encoding for p53 DNA-binding domain [29, 30]. The resulting proteins are full-length p53 proteins unable to bind DNA and to transactivate genes eliciting anti-tumoral activities [31]. Some mutant forms of p53 protein not only lose the wild-type activity but also acquire new functions that promote tumorigenesis [32–34]. Mutant p53 proteins can exert this gain of function activity through different molecular mechanisms. Among them, the most known are those implying both the transcriptional activation of oncogenic target genes through the physical interaction with other transcription factors, and the binding and sequestering of tumor suppressor proteins, such as p73 and p63, and the consequent impairment of their tumor suppressor activities [35–38]. In addition to this evidence, we have also observed a new mechanism by which mutant p53 exerts its gain of function activity, in particular chemoresistance: the regulation of miRNAs expression [39, 40]. Donzelli *et al*., originally identified miR-128-2 as a direct transcriptional target induced by mutant p53His175 protein, a well known mutant form of p53 that exerts gain of function activity. miR-128-2 overexpression determined an increase in chemoresistance to different anticancer drugs (cisplatin, doxorubicin and 5-fluorouracil). miR-128-2 targets the transcriptional repressor E2F5 which in turn resulted in the accumulation of cytoplasmic p21 protein, a feature that is frequently associated with the anti-apoptotic effect of p21. Indeed, lung cancer cells overexpressing miR-128-2 exhibit an increased chemoresistance to commonly anticancer treatments [40].

Masciarelli *et al*., showed that mutant p53His175 protein is able to inhibit miR-223 expression at the transcriptional level both in breast and colon cancer cell lines. This happened through the formation of a protein complex involving mutant p53 proteins and the transcriptional repressor ZEB1 on the miR-223 promoter. This transcriptional inhibition determined an increase in cell resistance to cisplatin and 5-fluorouracil treatments through at least in part, the up-regulated expression of miR-223 target STMN-1, a well known oncoprotein that is over-expressed in different human cancers, whose regulation is essential for cell cycle progression. Indeed, STMN-1 silencing by miR-223 overexpression induced an increase in cdk1 phosphorylation on residues Thr14/Tyr15, a modification that inhibits cdk1 activity, blocking the entrance into the M phase [39].

In summary, these two studies identified two miRNAs that contrastingly contribute to mutant p53-dependent chemoresistance.

More recently, Ganci et al. contributed significantly to correlate p53 mutations, selective expression of miRNAs and clinical output of head and neck cancer patients [41]. In particular, miRNA expression profiling of 121 head and neck squamous cell carcinoma (HNSCC) samples and 66 normal counterparts, allowed the identification of a group of 12 miRNAs whose expression correlates with short recurrence-free survival and a group of 4 miRNAs that correlates with worse cancer-specific survival of patients carrying p53 mutations.

PTEN is another important tumor suppressor gene correlated to chemotherapeutic response. In fact, its inactivation is often involed in drug resistance. Different miRNAs have been shown to regulate tumor cell chemoresistance by targeting PTEN. miR-21has been reported to regulate PTEN expression and the phosphorylation of its downstream kinase Akt, determining an increased sensitivity to gemcitabine in malignant human cholangiocytes and to cisplatin in gastric cancer [42–45]. Recently, Zhao et al. have identified miR-221 as a mediator of osteosarcoma cells resistance to cisplatin by demonstrating its ability to downregulate PTEN expression. They also observed a strong inverse correlation between miR-221 expression and PTEN protein levels in osteosarcoma tissues [46]. In two different studies miR-214 has been shown to mediate resistance to cisplatin of ovarian cell cancer and to gefitinib of non-small cell lung cancer EGFR mutated cell lines through the targeting of PTEN expression [47, 48].

Liang and colleagues have shown the involvement of miR-19 in the regulation of multidrug resistance (MDR) of breast cancer cells through PTEN inhibition [49].

In contrast, Li and colleagues identified miR-22, a tumor suppressor miRNA, as a positive regulator of PTEN. In colon cancer cells carrying mutant p53 protein miR-22 enhanced cell sensitivity to paclitaxel treatments by promoting PTEN expression and the consequent inhibition of Akt phosphorylation [50].

Recently, Rao and colleagues identified miR-17-92 cluster as a mediator of chemoresitance in mantle cell lymphoma [51]. miR-17-92 cluster is able to activate PI3K/Akt pathway by targeting two different proteins, both of them involved in the inhibition of this pathway: PTEN and PHLPP2, a member of PP2A-type phosphatases family. PTEN acts by removing the activating lipid second messenger, while PHLPP2 acts by promoting the dephosphorylation of activated AKT. Moreover, the authors identified another miR-17-92 cluster target protein: BIM. It is one of the most potent pro-apoptotic BH3-only proteins, which binds to all pro-survival BCL2 family members with high affinity, thereby releasing BAX and BAK proteins, the critical downstream effectors of the BCL2-regulated pathway of apoptosis. miR-494 overexpression determined a reduction of BIM protein level and a consequent increase in TRAIL-resistance in non small cell lung cancer [52].

Yan and colleagues showed that in pancreatic cancer cells miR-17-5p induced an increase in chemoresistance to gemcitabine by targeting BIM 3’UTR.

BIM has also been shown to be a target of miR-10b in colorectal cancer cells. miR-10b-mediated downregulation of BIM expression determined chemoresistance to 5–fluorouracil [53].

Two recent lines of evidence revealed also Bcl2l, an anti-apoptotic protein of Bcl2 family, to be under miRNAs control. In particular, Wang and colleagues identified miR-214 as the mediator of increased chemosensitivity to cisplatin in cervical cancer cells due to its ability to directly counteract Bcl2l expression, determining an increased expression of Bax, caspase-9, caspase-8 and caspase-3 [54]. Qu and collaborators demonstrated that miR-195 was downregulated in doxorubicin-resistant colon cancer cells and that its suppression in parental cells caused a significant reduction of doxorubicin induced apoptosis due to its ability to downregulate Bcl2l protein expression. They also validated this evidence *in vivo* demonstrating a marked inverse correlation between miR-195 and Bcl2l expressions in doxorubicin-resistant and sensitive colon cancer tissues [55]. MiR-204 has recently been shown to be involved in cells chemo-sensitivity to anticancer drugs due to its inhibitory effect on Bcl2 expression. In particular, Ryan and colleagues demonstrated miR-204’s role in increased neuroblastoma cell sensitivity to cisplatin treatment [56]. Sacconi and collaborators profiled miRNAs expression in gastric cancer tissue by comparing the tumoral and the matched peritumoral samples and they identified miR-204 downregulation to be a prognostic factor for gastric cancer [56, 57]. MiR-204 ectopic expression significantly potentiated the apoptotic effects induced by either oxaliplatin or 5-fluorouracil on gastric cancer cell lines by reducing the expression of Bcl2 protein.

Both intrinsic and extrinsic apoptotic pathways result in the activation of caspase-3, the main apoptotic effector. It has also been demonstrated that let-7a exogenous expression in human squamous carcinoma and hepatocellular carcinoma cells increased the resistance to different therapeutic drugs, such as interferon-gamma, doxorubicin and paclitaxel by targeting caspase-3 [58]. More recently, Quintavalle and colleagues identified miR-30 b/c and miR-21 to be upregulated in TRAIL-resistant cells [59]. By further *in vitro* investigations they identified the molecular mechanism by which miR-30b/c and miR-21 determined a TRAIL-resistant phenotype. MiR-21 has been shown to inhibit caspase-3 expression by directly binding to its mRNA 3’UTR, while miR-30 has been demonstrated to bind the 3’UTR of TAp63, inducing its translational repression, that in turn determining the downregulation of different genes involved in apotosis-control, such as TRAIL receptors genes.

Miller and colleagues showed that miR-221 and miR-222 contribute to tamoxifen resistance of breast cancer cells by targeting p27 ^kip^[60]. They also observed a higher expression of miR-221 and miR-222 in HER2/neu-positive primary human breast cancer tissues (known to be resistant to endocrine therapy) compared with HER2/neu-negative tissue samples.

### miRNAs in the control of drug uptake

The balance between the entering and exiting of a drug is fundamental within the cancer cell. Drugs enter in a cell in different ways, from diffusion to endocytosis or through the use of a protein functioning as a transporter. Decreasing drug concentration could be affected by a mutation that modify or eliminate the functional activity of cell surface molecules. For example, cells resistant to metotrexate have commonly mutated folate binding proteins [61].

Alterations in drug efflux were widely studied and considered one of the primary causes of multidrug resistance (MDR) [62]. In particular, overexpression of ABC (ATP binding cassette) super family of transporters by tumor cells is closely linked to chemoresistance [63]. ABC transporters are transmembrane proteins that through ATP hydrolysis transport drugs outside of the cells against a drug gradient. Three ABC proteins were mainly described for most MDR in humans: P-glycoprotein (P-gp), MDR-associated protein 1 (MRP1) and breast cancer resistance protein (ABCG2) [64].

Different studies have shown that also the expression MDR-related proteins is regulated by miRNAs in different tumor types. Bao et al. observed a decreased expression of miR-298 which paired with increased expression of P-gp by comparing miRNAs expression between doxorubicin-sensitive and -resistant breast cancer cells [65]. They found that miR-298 directly bound to P-gp 3’UTR causing its downregulation and consequent increase of breast cancer cell sensitivity to doxorubicin.

Recently, miR-19a/b have been implicated in multidrug resistance mechanism [66]. The expression of these miRNAs was upregulated in a multidrug-resistant gastric cancer cell lines and in turn determined increased P-gp expression levels. This led to an accelerated drug efflux through the modulation of PI3K/Akt pathway. MiR-451 and miR-27a have also been shown to be directly correlated to P-gp overexpression in multidrug-resistant ovarian and cervical cancer cell lines [67].

Liang and collaborators have identified miR-326 as an inhibitor of multidrug resistant phenotype due to its ability to directly downregulate MRP1 expression levels. MiR-520 h in hematopoietic stem cells, miR-519c in A459 lung cancer cell line and miR-328 in breast cancer cells, have been shown to directly regulate the expression of BCRP [68–70].

### miRNAs in the control of drug metabolism

Tumor cells can acquire resistance to a specific drug by altering pathways involved in drug metabolism. The super family of cytochrome p450 (CYP) enzymes play a critical role in this important process. The expression of some of its members is regulated by miRNAs. In particular, Tsuchiya and collaborators identified miR-27b as a regulator of CYP1B1 protein expression. CYP1B1 is a key member of the CYP family, is highly expressed in tumor tissues and mediates the metabolism of a wide range of drugs [71]. MiR-27b targeted CYP1B1 expression by binding its 3’UTR. Inverse correlation between CYP1B1 and miR-27b expression levels in breast cancer tissues has been evidenced; thereby suggesting that CYP1B1 overexpression in tumor tissues could be a consequence of miR-27b downregulation.

CYP3A4 is another key enzyme involved in drug metabolism. It is mainly expressed in hepatic and intestinal tissues. Takagi and colleagues identified miR-148a as an indirect regulator of CYP3A4 expression levels [72]. They showed that mir-148a targeted the 3’UTR of pregame X receptor (PXR) that acts as a transcriptional regulator of different genes among which CYP3A4. The inhibitory effect of miR-148a on PXR expression resulted in a decreased induction of CYP3A4 in liver cancer.

### miRNAs as predictive biomarkers of chemotherapeutic response

In light of the advances in miRNA detection techniques, such as the development of microarrays, it is now possible to have a clear picture of the miRNA expression profile for many types of cancer and to correlate it to a particular type of anticancer agent response. All these studies have been performed both in cancer cell lines and in tumor tissues (see Table 2).

**Table 2:** **miRNAs profiling studies correlated to drugs response**

Cancer type	n° of deregulated miRNAs	Chemoteraupeutic drugs	Reference
**Breast cancer cell lines** chemoresistant vs chemosensi.ve	137 (63 up, 75 down)	Doxorubicine	[76]
**Breast cancer cell lines** chemoresistant vs chemosensi.ve	15 (8 up, 7 down)	Tamoxifen	[61]
16 **ovarian cancer cell lines**	27 (18 up, 9 down)	Cisplatin, doxorubicine, topotecan, paclitaxel, docetaxel, gemtabicine	[77]
**Ovarian cancer cell lines** chemoresistant vs chemosensi.ve	6 (3 down in all resistant cell lines)	Cisplatin, paclitaxel	[78]
**Ovarian cancer cell lines** ALDH + vs ALDH-chemoresistant vs chemosensitive ALDH + cell lines	6 up	Paclitaxel	[79]
**Lung cancer cell lines** chemoresistant vs chemosensi.ve	7 (5 up, 2 down)	TRAIL	[80]
**Prostate cancer cell lines** chemoresistant vs chemosensi.ve	10 (5 up, 5 down)	Cisplatin, docetaxel	[81]
**Gastric cancer sapmles** pre-treated vs normal	58	Cisplatin, 5-fluorouracile	[82]

For instance, Kovalchuk and colleagues, found a signature of 137 deregulated miRNAs (63 upregulated and 75 downregulated) by comparing doxorubicine-resistant and doxorubicine –sensitive breast cancer cell lines [73].

Another study carried out on a tamoxifene-resistant breast cancer cell line, performed by Miller and collegues, led to the identification of a signature of 15 deregulated miRNAs: 8 upregulated miRNAs (miR-221, miR-222, miR-181, miR-375, miR-32, mir-171, miR-213, miR-203) and 7 downregulated miRNAs (miR-342, miR-489, miR-21, miR-24, miR-27, miR-23, miR-200) [60].

Boren and colleagues performed a miRNA microarray analysis on 16 different ovarian cancer cell lines [74]. In their study, they found 27 miRNAs highly associated with response to one or more of the six chemoterapeutic agents commonly used in ovarian cancer therapy (cisplatin, doxorubicin, topotecan, paclitaxel, docetaxel, and gemcitabine): 18 miRNAs showed increased expression (miR-213, miR-181b, miR-181a, let 7e, miR-520f, miR-21, miR-502, miR-514, miR-371, miR-99b, miR-518c-AS, miR-515-5p, miR34b, miR-431, miR126, miR-23b, miR-381, miR-340) and 9 miRNAs decreased expression (miR-518c, miR-132, miR-330, miR-339, miR-142-5p, miR-29c, miR-331, miR-185, miR-106a) with increasing resistance to individual drugs. Seven out of 27 miRNAs were associated with response to more than one drug (miR-213, miR181a and miR 181b to doxorubicin and gemcitabine; miR-99b and miR-514 to docetaxel and paclitaxel; miR-518c-AS to docetaxel and topotecan; miR-520f to doxorubicin and cisplatin). The authors also performed a cross-analysis between the 27 miRNAs and their predicted mRNA targets. They found 53 genes that were previously shown to be predictive of chemotherapy response.

In another miRNA profiling study, Sorrentino and colleagues further investigated the role of miRNAs in ovarian cancer chemoresistance by analayzing the miRNA expression in a panel of paclitaxel- (A2780TAX, A2780TC1 and A2780TC3) and cisplatin-resistant (A2780CIS) ovarian cancer cells [75]. They described a signature of six miRNAs (let-7e, miR-30c, miR-125b, miR-130a and miR-335) deregulated in all the resistant cell lines. In particular, miR-30c, miR-130a and miR-335 were found to be downregulated in all the resistant cell lines, suggesting that they play an important role in chemoresistance. Let-7e resulted to be upregulated only in A2780TAX cells and to be downregulated in the other cell lines. Conversely, miR-125b expression was downegulated only in A2780TAX and upregulated in the other cell lines. One of the most important implications of these miRNAs in chemoresistance was also confirmed by the validation of a target gene miR-130a, M-CSF, whose downregulation is a wellknown resistance factor in ovarian cancer.

More recently, Park and colleagues carried out a miRNA profiling study by isolating the population of ALDH1(+) cells, a potential cancer stem cell marker, in different ovarian cancer cell lines (SKOV3, A2780, and OVCAR 3), chemoresistant sublines (SKpac-12,-16,-17, A2780pac, and A2780cis), and primary tumor cells (SCN-1–7) [76]. By comparing the ALDH1(+) cells with the ALDH1(−) cells they identified six miRNAs significantly overexpressed (miR-424, miR-346, miR-503, miR-27a, miR-23b, and miR-27b). Then, they investigated the impact of these six ALDH1(+)-associated miRNAs on chemoresistance by comparing their expression levels between chemoresistant SKpac sublines and chemosensitive SKOV3 cell line. What they found is higher expression levels of both six miRNAs and ALDH1 protein in SKpac sublines. They validated these results in ovarian cancer samples by finding greater expression levels of miR-23b, mir-27b, miR-424 in the chemoresistant group.

Another study conducted by Garofalo and colleagues, identified a signature of miRNAs involved in TRAIL resistance of human non-small cell lung cancer [77]. In particular, they performed a miRNA profiling analysis in four NSCLC cell lines that present a different grade of resistance to TRAIL treatments: CALU-1 (TRAIL-resistant), A459 and A549 (semi-resistant) and H460 (TRAIL-sensitive). With this analysis, they found a signature of five significantly overexpressed miRNAs (miR-222, miR-100, miR-221, miR-125b and miR-15b) and two downregulated ones (miR-9 and miR-96) in resistant NSCLC cells. They further investigated the role of miR-221 and miR-222 in TRAIL resistance by looking at two of their target genes, Kit and p27^kip1^, highlighting the role of p27^kip1^ inhibition as mediator of both miR-221 and miR-223 TRAIL-resistance.

Bhatnagar and collegues performed the same study in the context of prostate cancer by comparing miRNA expression profiling in two prostate cancer cell lines: WPE1-NA22 (early cancer) and WPE1-NB26 (advanced cancer and more resistant cells) [78]. From this type of analysis, they found five downregulated and five upregulated miRNAs in WPE1-NB26 cells. They focused their attention on the two most downregulated ones: miR-200 and miR-31, whose deregulation was also validated in other malignant prostate cancer cell lines. Through *in vitro* experiments they showed that miR-205 and miR-31 controlled apoptosis in prostate cancer cells by targeting antiapoptotic proteins Bcl-w and E2F6.

Kim and colleagues succeeded in identifying a miRNA signature able to predict clinical resistance to cispaltin/fluorouracil treatment of gastric cancer [79]. They first compared miRNA profiles from 90 pre-treated gastric cancer samples with miRNA expression data taken from 34 normal gastric mucosal biopsy samples obtained from healthy donors. Subsequently, they used time to progression (TTP) as a clinical indicator for chemotherapy response. This parameter led to identifying a signature of 58 miRNAs. The overexpression of 30 out of 58 miRNAs was associated with delayed TTP whereas the overexpression of the remaining 28 miRNAs was associated with a more rapid TTP.

These types of study design, similar to the ones just mentioned, are very innovative because they contribute to assign to miRNAs the role of biomarkers for predicting cancer therapy outcomes, which in turn lead to identifying in individual patients the outcome of their cytotoxic chemotherapy.

### miRNA therapeutic implications

The discovery of miRNAs adds another layer of gene regulation that is subjected to change in human disease, including cancer. Aberrantly expressed miRNAs play key roles in the development of human disease, and consequently the correction of these miRNA deficiencies using antagomirs or mimic synthetic miRNA may provide a therapeutic benefit. Indeed, miRNAs frequently acquire a gain or a loss of function in cancer thus playing a causative role in the development of the disease [80, 81]. Aberrant regulation of miRNAs is manifested by differential miRNA expression in the tumor tissue compared to the normal adjacent tissue as it has been described for instance, for miR-204 in gastric cancer, miR-10b* in breast cancer and miR-145 in mesothelioma [57, 82, 83]. miRNA deregulation can be the consequence of genomic rearrangements or altered methylation statuses of their respective promoter regions. The functional consequence of miRNA deregulation became evident as the introduction or repression of a single miRNA can effectively contribute to tumorigenesis or tumor progression. Several functional studies using cultured cancer cells and *in vivo* cancer models have identified miRNAs as conventional tumor suppressors or oncogenes. The therapeutic application of miRNAs involves two strategies. One strategy is directed toward a gain of function which aims to inhibit oncogenic miRNAs by using miRNA antagonists, such as anti-miRs, locked-nucleic acids (LNA), or antagomiRs. These miRNA antagonists are oligonucleotides with sequences complementary to the endogenous miRNA. They carry chemical modifications that enhance the affinity for targeting miRNA and trapping the endogenous miRNA in a configuration that is unable to be processed by RISC, or alternatively leads to degradation of the endogenous miRNA. The second strategy, miRNA replacement, involves the reintroduction of a tumor-suppressor miRNA mimic to restore a loss of function [84]. The restoration of tumor suppressor miRNAs might increase the efficiency of conventional cancer chemotherapy. The inefficient delivery or degradation of the miRNA requires multiple doses of replacement of a given tumor suppressor miRNA. Alternatively, viral vectors encoding short hairpin RNAs that are processed by the cell into mature miRNAs could be used to transduce target cells [81, 85, 86]. Viral delivery of miRNAs can be optimized to achieve a specific and continuous level of expression. Short hairpin RNAs that are more pre-miRNA-like or authentic pre-miRNAs themselves will minimize toxicity while at the same time retain potency for their intended targets [87–89]. miRNAs provide a new opportunity because, unlike proteins, miRNA mimics are substantially smaller, they just have to enter the cytoplasm of target cells to be active, and can be delivered systemically using methods and technologies that are also used for siRNAs. There are several other key observations supporting the concept of miRNA replacement therapy. These include: (i) the majority of differentially expressed miRNAs is suppressed in tumor tissue relative to normal tissues, indicating that the probability for miRNAs acting as tumor suppressors than oncogenes is greater [90]; and (ii) inhibition of endogenous miRNA processing induces oncogenic transformation and increases tumorigenesis, suggesting that the tumor suppressive role of miRNAs prevails over an oncogenic role [91]. Another advantage of miRNA mimics is the fact that nonspecific off-target effects are unlikely as miRNA mimics are expected to behave like the natural counterpart in the proper miRNA-mRNA interactions. The strongest rationale for exploring the therapeutic potential of miRNAs, however, is based on the observation that a single miRNA can regulate multiple oncogenes and oncogenic pathways that are commonly deregulated in cancer. In addition, recent data implicating miRNAs in self-renewing tumor-initiating cancer cells (cancer stem cells) may significantly extend the scope of miRNA mimics and may suggest that miRNAs can become valuable tools in eliminating cancer cells frequently associated with chemoresistance, metastasis, and recurrence [84, 92, 93].

## Conclusions and future prospectives

Chemoresistance still remains the greatest difficulty to overcome in cancer therapy. The discovery of miRNAs has opened up new opportunities in understanding the molecular mechanisms involved in different diseases and in developing new and more efficient therapies.

As mentioned before, miRNAs are involved in the regulation of different molecular pathways and the capability of modulating the expression of a plethora of genes makes them master regulators of multiple networks, so that even slight modulations in their expression levels may determine significant changes in cancer cell fate.

In this context, the research of potential prognostic power of miRNAs is still at an early stage. Up to now, miRNA signatures have proved to be very useful for classifying different tumors and helpful for diagnosing different cancer types.

**Figure 2:**
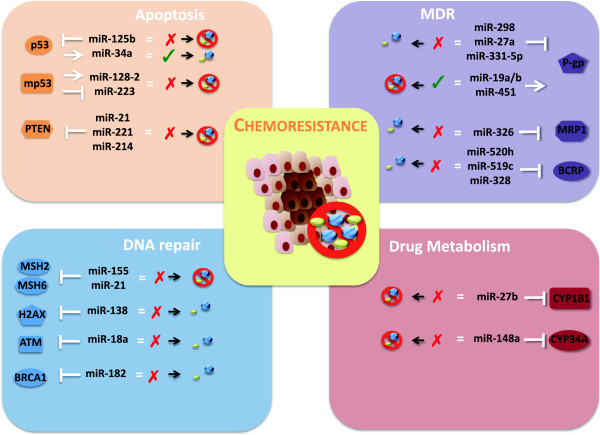
**Schematic representation of the mechanisms of action of chemoresistance-associated miRNAs.** MicroRNAs have been demonstrated to be involved in the modulation of chemoresistance of cancer cells trough the regulation of the expression of different target genes involved in the control of apoptosis, or multi drug resistance, or DNA repair or drug metabolism.

As it has been extensively discussed in this review, miRNAs, in regards to tumor cell sensitivity response to different drug treatments, may have a positive or a negative effect, according to the type of drug, cellular context and molecular pathway they control (Figure 2). All the evidence gathered so far makes it possible to think of therapy development, that include the use, alone or in combination with anticancer drugs, of molecules that antagonize the expression of specific miRNAs, such as antagomirs, or that restore or strengthen the function of miRNAs with an inhibitory effect on the expression of pro-chemoresistance gene, such as mimics. These kinds of molecules have still not been introduced in clinical trials, except for one: the case of Miravirsen, that is the first miRNA-targeted drug to enter clinical trials for the treatment of Hepatitis C virus [94]. This drug has successfully reached the 2a phase of a clinical study and it consists in a LNA (Locked Nucleic Acid) against miR-122, the miRNA necessary for Hepatitis C virus replication. This study provides much promise and high hopes for successfully introducing miRNAs in therapies planned for different diseases among which certainly cancer.

It is also noteworthy to stress the opportunity suggested by miRNA profiling studies in chemoresistant models to use these molecules as predictive biomarkers of therapy outcomes, in order to develop more effective personalized treatments.

Thus, we can conclude that in such small molecules great promise is hidden for the future of cancer therapy.

## Abbreviations

miRNA: microRNA
HCC: Human hepatocellular carcinoma
MRP-1: Multidrug resistance-associated protein 1
MDR: Multidrug resistance
MDR-1: Multidrug resistance 1.
